# Editorial: Psychological Responses to Violations of Expectations

**DOI:** 10.3389/fpsyg.2017.02357

**Published:** 2018-01-23

**Authors:** Mario Gollwitzer, Anna Thorwart, Karin Meissner

**Affiliations:** ^1^Department of Psychology, Philipps University of Marburg, Marburg, Germany; ^2^Institute of Medical Psychology, Ludwig-Maximilians-Universität München, Munich, Germany; ^3^Division of Health Promotion, Department of Social Work and Health, Hochschule Coburg, Coburg, Germany

**Keywords:** expectation violation, associative learning, clinical psychology, social psychology, individual differences

The general aim of this Research Topic was to collect and systematize theoretical approaches and latest empirical evidence on expectation violations, or, more precisely, on how individuals cope with such violations. This question is relevant from a basic science as well as from an applied perspective. Sometimes, expectations persist even in the face of disconfirming evidence. For instance, social stereotypes remain sticky even after confronting stereotype-inconsistent exemplars, and fear-related expectations are hard to tackle in the course of psychotherapeutic interventions. What are the psychological mechanisms underlying a sustainable change of expectations vs. a persistence of expectations in the face of disconfirming evidence?

The 21 articles collected in the present Research Topic shed more light on this question. As guest editors of this Topic, we were glad to receive papers from so many sub-disciplines of psychology, including clinical psychology (Corsi and Colloca; Kube et al.; Rief and Petrie; Laferton et al.), social/personality psychology (Krueger et al.; Mieth et al.; Song and Zuo; Süssenbach et al.; Wesselmann et al.), learning psychology (Bustamante et al.; Griffiths et al.; Janssens et al.; Kemper and Gaschler; Thorwart and Livesey), cognitive psychology (Dötsch et al.; Foerster), and neurosciences (Angel and Seitz; D'Astolfo and Rief; Nasser et al.), and one paper even builds a bridge to political science (Öllinger et al.).

These papers also cover a broad range of methodological approaches, from theoretical discussion (e.g., Öllinger et al.; Angel and Seitz) via highly controlled lab studies (e.g., Foerster) and surveys (e.g., Sattler and Christiansen) to meta-analyses (e.g., D'Astolfo and Rief). The diversity of specific research questions, theoretical approaches, and methodological strategies is enormous and shows how prevalent expectation violations are and how relevant a psychological model for people's psychological responses to these expectations actually is.

That said, a common theoretical framework on how individuals process and deal with expectation violations is missing. Such a framework would be helpful to (1) establish a common language with properly defined concepts that can be usefully applied to psychological research on expectation violations in different areas, (2) describe the cognitive, affective, and social processes involved in individuals' responses to expectation violations, and (3) explain these responses psychologically. Such a model should not only be applicable to neuroscientific, but also to cognitive and social psychological approaches.

One model that we think may be helpful in that regard is the ViolEx Model (Rief et al., 2015). The ViolEx model defines expectations as conditional predictions about future events (or “if-*X*-then-*Y*” hypotheses) that may be changed or maintained in the face of disconfirming evidence (i.e., if an event or stimulus *X* is followed by a non-expected outcome $\bar{Y}$). The model differentiates between generalized expectations (e.g., “Whenever other people ask me for help, their intention is to exploit me”) and situation-specific, conditional predictions (e.g., “If I lend this book to my neighbor, he will never bring it back”). In general, only situation-specific predictions (but not generalized expectations) can be directly falsified empirically. If a specific prediction turns out to be correct and the expected outcome occurs, the model predicts that one's generalized expectation is reinforced or stabilized. Expectation violations, on the other hand, do not necessarily result in a change of one's generalized expectation.

Whether expectation change or rather expectation maintenance occurs in a given situation depends on the specific psychological process that is operating. The ViolEx model specifies three of these “coping” processes: accommodation, assimilation, and immunization^1^. Technically speaking, these processes mediate the effect of expectation violations on expectation change vs. maintenance.

*Accommodation* refers to mechanisms by which individuals adjust their expectation so that it fits to the (unexpected) outcome. Thus, accommodation is the process that underlies expectation change in the context of expectation-inconsistent outcomes and corresponds to what is generally referred to as learning (Thorwart and Livesey).

*Assimilation* refers to mechanisms by which individuals actively remove any future discrepancies between their expectations and expectation-inconsistent outcomes. This strategy includes (a) avoiding expectation-inconsistent outcomes (e.g., “fear avoidance” in clinical psychology; cf. Vlaeyen and Linton, 2012), and/or (b) actively contributing to a higher likelihood of expectation-consistent outcomes (i.e., “self-fulfilling prophecies;” Stinson et al., 2011; Hechler et al., 2016). Thus, individuals create situations that confirm their current expectations and reduce the effect of an expectation violation.

*Immunization* refers to mechanisms by which individuals minimize the potential impact of discrepant information on their expectations in a given situation. In the case of “data-oriented immunization,” individuals devalue discrepant information (e.g., denying the data or doubting its validity). In the case of “concept-oriented immunization,” individuals reframe the conceptual meaning of their expectation so that former discrepant information is no longer diagnostically valid (cf. Greve and Wentura, 2010). For instance, studies from social psychology show that confronting people with stereotype-inconsistent out-group exemplars does not necessarily change their stereotypes; stereotype-inconsistent exemplars are often “subtyped” as atypical exemplars of their respective group (Yzerbyt and Carnaghi, 2007). Thus, subtyping is a form of immunization. Possible implications of such immunization processes are far-reaching and may even comprise misguided political decision making (Öllinger et al.).

Taken together, the ViolEx model assumes that organisms can react to expectation violations by following one of three routes (i.e., accommodation, assimilation, immunization), and only one of these routes (i.e., accommodation) actually leads to a sustainable change in existing expectations.

The ViolEx model further predicts that (a) direct experiences, (b) social (and cultural) influences, and (c) individual differences influence which route an organism “chooses” to follow. In other words, each of these three factors influences the probability with which accommodation, assimilation, or immunization occurs. Technically speaking, these factors moderate the effect of expectation violations on expectation maintenance vs. change.

*Direct experiences* include current situational expositions or prior experiences with *X* and/or *Y* and other stimuli. For example, Griffiths et al. explore whether creating a strong expectation by presenting two separate predictive events simultaneously (*X*_1_ and *X*_2_) results in more accommodation and Bustamante et al. investigate the modulatory impact of different “reminder cues” during expectation-consistent and expectation-inconsistent situations on processing these situations. Other findings show that expectations are changed more rapidly when there were only few expectation violations experienced before (e.g., Thorwart et al., 2017). A factor that it also relevant in this regard (and which is has not been explicitly incorporated into the ViolEx model) is how an initial expectation has been generated in the first place. As Kemper and Gaschler argue, self-generated expectations may be more resistant to change than cue-induced expectations. In line with this argument, Janssens et al. show how pre-existing conceptual beliefs shape expectations generated by a cue, and Thorwart and Livesey offer three solutions for how influences of such information can be incorporated into existing learning models.

*Social influences* include peers, significant others, the media, or any other social or cultural factors. They are particularly relevant in cases of *social* expectations; for instance, expectations about being socially included (Wesselmann et al.), about others' actions in a social dilemma (Krueger et al.), or about other people's trustworthiness (Mieth et al.; Süssenbach et al.). Using the latter as an example, Krueger et al. show that social distance to others is (negatively) correlated with people's expectations that they will cooperate. Finally, the strength of culturally shared stereotypes strongly predicts the stickiness of expectations (Song and Zuo).

*Individual differences* include personality traits as well as biological/genetic factors. For instance, victim sensitivity—individuals' disposition to react toward injustice at one's own disadvantage (Schmitt et al., 2005)—is associated with a latent (“generalized”) expectation of other people being selfish and untrustworthy (Gollwitzer et al., 2013). As Süssenbach et al. show, victim-sensitive individuals have a better source memory for events in which this latent expectation has been violated. Regarding biological/genetic factors, research shows that personality traits that are related to genetic differences in dopaminergic and serotonergic processes may be critical for inter-individual differences in processing reward-prediction errors (e.g., Müller et al., 2014), which is also true for dopamine- and extraversion-related gene-variants (Müller et al., 2011).

The ViolEx model is a useful framework for different approaches to investigations of expectation violations. This does not mean that it is the best of all possible models. In fact, there are other models (such as the Credition model portrayed by Angel and Seitz), and, of course, the ViolEx model may need to be adapted to represent the specific aspects of a particular research area. In this vein, Rief and Petrie show how the ViolEx model can be adapted to research on Placebo/Nocebo effects in clinical psychology. Furthermore, the ViolEx model is currently silent on the neuropsychological implementation of its variables and processes as well as its links to other relevant research, for example, on the dopamine prediction error (Nasser et al.).

This Research Topic shows that for scholars in different psychological research areas, investigating individuals' reactions to violations of expectations is a fascinating endeavor. Many pieces of the puzzle have been collected, but not yet put together in an integrative fashion. We think that this Research Topic facilitates structuring research and theory-building and advances models and theoretical frameworks such as the ViolEx model.

## Author contributions

All authors listed have made a substantial, direct and intellectual contribution to the work, and approved it for publication.

### Conflict of interest statement

The authors declare that the research was conducted in the absence of any commercial or financial relationships that could be construed as a potential conflict of interest.

## Footnotes

^1^These terns are borrowed from research on coping with age-related stressors (Brandtstädter and Greve, 1994; Rothermund and Brandtstädter, 2003). Since this research does not talk so much about the change vs. persistence in expectations (but rather about the change vs. persistence of goals, plans, and self-concepts), the terms have a slightly different meaning in the present context.
